# Identification of extensive drug resistant *Pseudomonas aeruginosa* strains: New clone ST1725 and high-risk clone ST233

**DOI:** 10.1371/journal.pone.0172882

**Published:** 2017-03-02

**Authors:** Pamela Aguilar-Rodea, Gerardo Zúñiga, Benjamín Antonio Rodríguez-Espino, Alma Lidia Olivares Cervantes, Ana Estela Gamiño Arroyo, Sarbelio Moreno-Espinosa, Daniela de la Rosa Zamboni, Briceida López Martínez, María del Carmen Castellanos-Cruz, Israel Parra-Ortega, Verónica Leticia Jiménez Rojas, Juan Carlos Vigueras Galindo, Norma Velázquez-Guadarrama

**Affiliations:** 1 Posgrado en Ciencias Quimicobiológicas, Escuela Nacional de Ciencias Biológicas, Instituto Politécnico Nacional, México City, México; 2 Laboratorio de Infectología, Hospital Infantil de México Federico Gómez, México City, México; 3 Laboratorio de Variación Biológica y Evolución, Departamento de Zoología, Escuela Nacional de Ciencias Biológicas, Instituto Politécnico Nacional, México City, México; 4 Laboratorio de Investigación y Diagnóstico en Nefrología y Metabolismo Mineral Óseo, Hospital Infantil de México Federico Gómez, México City, México; 5 Departamento de Infectología, Hospital Infantil de México Federico Gómez, México City, México; 6 Departamento de Epidemiología, Hospital Infantil de México Federico Gómez, México City, México; 7 Subdirección de Servicios Auxiliares y de Laboratorio, Hospital Infantil de México Federico Gómez, México City, México; 8 Departamento de Laboratorio Clínico, Hospital Infantil de México Federico Gómez, México City, México; Beijing Institute of Microbiology and Epidemiology, CHINA

## Abstract

Several microorganisms produce nosocomial infections (NIs), among which *Pseudomonas aeruginosa* stands out as an opportunist pathogen with the capacity to develop multiresistance to first-choice antibiotics. From 2007 to 2013, forty-six NIs produced by *P*. *aeruginosa* were detected at a pediatric tertiary care hospital in Mexico with a significant mortality rate (17.39%). All isolates (n = 58/46 patients) were characterized by evaluating their response to several antibiotics as panresistant (PDR), extensively resistant (XDR), multiresistant (MDR) or sensitive (S). In addition, all isolates were typified through multilocus sequencing of seven genes: *acs*A, *aro*E, *gua*A, *mut*L, *nuo*D, *pps*A and *trp*E. Furthermore, to establish the genetic relationships among these isolates, we carried out a phylogenetic inference analysis using maximum likelihood to construct a phylogenetic network. To assess evolutionary parameters, recombination was evaluated using the PHI test, and the ratio of nonsynonymous to synonymous substitutions was determined. Two of the strains were PDR (ST1725); 42 were XDR; four were MDR; and ten were S. Twenty-one new sequence types were detected. Thirty-three strains exhibited novel sequence type ST1725. The ratio of nonsynonym to synonym substitutions was 1:1 considering all genes. Phylogenetic analysis showed that the genetic relationship of the PDR, XDR and MDR strains was mainly clonal; however, the PHI test and the phylogenetic network suggest that recombination events occurred to produce a non-clonal population. This study aimed not only to determine the genetic diversity of clinical *P*. *aeruginosa* but also to provide a warning regarding the identification and spreading of clone ST1725, its ability to cause outbreaks with high mortality rates, and to remain in the hospital environment for over seven years. These characteristics highlight the need to identify clonal outbreaks, especially where high resistance to most antibiotics is observed, and control measures are needed. This study also represents the first report of the PDR ST1725.

## Introduction

*P*.*aeruginosa* is an important opportunistic pathogen worldwide, causing infections in patients with immunosuppression [1]. The presence of *P*. *aeruginosa* in hospitals is due to its great physiological capacity to use a wide variety of organic substrates as carbon and nitrogen sources, allowing its survival for long periods of time [2]. *P*. *aeruginosa* also shows an advanced ability to resist antibiotics due to mutations and the acquisition of new genes that promote the development of MDR (nonsusceptibility to at least one agent in three or more antimicrobial categorie), XDR (nonsusceptibility to at least one agent in all but two or fewer categories) and PDR (nonsusceptibility to all antimicrobial agents in all categories), which are characteristics that induce high mortality rates due to the limited choice of antibiotics available for treatment [3,4,5].

Colonization of patients with *P*. *aeruginosa* is caused by strains acquired from the environment, which may lead to persistent infections and the development of mechanisms of resistance [3,6]. Both of these attributes make this bacteria highly transmissible and virulent, favoring inter-patient dissemination and increasing its evolutionary potential[4,7].

In recent years, several reports have called attention to the emergence and spreading of clones of *P*. *aeruginosa* showing MDR and XDR characteristics with high epidemic risk in various hospitals worldwide[8]. For example, clones with sequence types ST111, ST175, ST235, ST253 and ST274 and the Liverpool epidemic strain (LES-1) initially appeared at one location and were later found to have undergone significant global spreading, causing high mortality rates[3,4,6,8].

*P*. *aeruginosa* is reported to be the main cause of nosocomial infections in the USA (7.1%) and European countries (8.9%)[4,9]. In Mexico, the “Sistema de Vigilancia Epidemiológica Hospitalaria del Instituto Mexicano del Seguro Social” reported an incidence rate of 19.9% in 2013 representing an increase of 6.9% compared with the rate of cases reported in 2011[10].

Population genetics studies of microorganisms of clinical importance have been essential for understanding and predicting their evolution. Several authors have reported that *P*. *aeruginosa* exhibits a non-clonal epidemic population structure and that much of its genetic variation is the result of recombination events[3,4,11]. This variation has allowed some strains to adapt to the hospital environment and prevail for long periods of time at these sites. For example, Deplano *et al*. (2005) conducted molecular characterization of an MDR *P*. *aeruginosa* clone that caused an outbreak in an intensive care unit in a hospital in France, which was isolated from the sink of a colonized patient and from the hands of a nurse[12]. García-Castillo *et al*. (2011) showed that the populations of carbapenem-nonsusceptible *P*. *aeruginosa* present in 16 Spanish hospitals were highly diverse; in addition, they found that the ST175 clone was present in 10 of the 16 hospitals[13]. Cholley *et al*. (2011) reported the presence of widespread MDR *P*. *aeruginosa* clones in French hospitals, suggesting that the MDR condition in successful clones was due to antibiotic pressure, allowing their spreading in hospitals[14]. López-Causapé *et al*. (2013) demonstrated the prevalence of 13 different clones of *P*. *aeruginosa* in a Spanish hospital after monitoring ten cystic fibrosis patients for over eight years; in some of these patients, coexistence of different clones was observed, and a highly transmissible and persistent clone was identified among them, resulting from the accumulation of resistance over time[7].

It is widely accepted that the survival and persistence of *P*. *aeruginosa* in hospitals are explained by the rise of adaptations associated with increasing resistance to certain antibiotics that are routinely used for the control and eradication of the microorganism[6]. It is therefore very likely that high-risk clones or even clonal complexes are circulating worldwide and producing outbreaks with high mortality rates[4]. In addition, the possibility of patients becoming ill or even dying due to a *P*. *aeruginosa* infection that was not the reason for their admission to the hospital is highly probable because infection is favored by the quality of care and cleaning conditions. Thus, surveillance protocols have been developed in all health institutions to prevent and control nosocomial infections NIs worldwide.

The epidemiological investigation of microorganisms is considered a support tool that ensures the proper functioning of medical services. In fact, the molecular characterization of these microorganisms has been crucial for identifying their evolutionary processes[12,14–16] and how important characteristics such as resistance to antibiotics change over time within the hospital environment.

At a pediatric tertiary care hospital in Mexico City in 2007, *P*. *aeruginosa* clones emerged, spread and produced high infant mortality rates. Different isolates of these clones were biochemically characterized and monitored within the hospital until 2013. This analysis revealed the presence of sensitive, multiresistant, extremely resistant and panresistant strains among these isolates[17]. However, no study was conducted during this period to understand the genetic relationships and evolutionary dynamics of these strains. The aim of this study was to analyze the changes in susceptibility, genetic relationships and evolutionary dynamics of *P*. *aeruginosa* clones at the Hospital Infantil de México Federico Gómez (HIMFG). This study provides the background of the extremely resistant and panresistant clone ST1725; we also report the presence of clone ST233 (previously reported in other parts of the world) with panresistance characteristics.

## Materials and methods

### Bacterial isolates

*P*. *aeruginosa* strains were isolated in the Departament of Central Laboratory of the HIMFG, from patients with nosocomial infections documented by the Epidemiology department under the written informed consent HIM-LC-RC-PR.01-RE.01. The ethics committee (Dr. Luis Jasso Hernández), research committee (Dr. Onofre Muñoz Hernández) and biosafety committee (Dra. Herlinda Vera Hermosillo) of the Hospital Infantil de México Federico Gómez reviewed and approved the project [HIM/2015/023]

The *P*. *aeruginosa* strains were grown on blood agar plates at 37°C for 24 h. Identification was performed using conventional methods, including analyses of colony morphology, microscopic morphology, oxidase +, β hemolysis, pigment production (pyocyanin and pyoverdin) and characteristic odor, the Kliger biochemical test and tests of growth in Cetrimida at 42°C and gas production from nitrates. Furthemore, the VITEK2® automated system (Biomerieux Marcy l'Etoile, France) equipment was employed.

### Susceptibility profile

The antimicrobial susceptibility of isolated *P*. *aeruginosa* strains was tested using Minimum Inhibitory Concentrations (MIC), employing the agar dilution method as described by Clinical and laboratory Standards Institute (CLSI 2016) [18]. The antibiotics used were: Aminoglycosides: Gentamicin (GEN)(Sigma-Aldrich, St. Louis, MO), Tobramycin (TOB)(MP Biomedicals, Solon, OH), Amikacin (AK) (Sigma-Aldrich, St. Louis, MO); Carbapenems: Imipenem (IMI) (Sigma-Aldrich, St. Louis, MO), Meropenem (MEM) ((Sigma-Aldrich, St. Louis, MO); Cephalosporins: Ceftazidime (CAZ)(Sigma-Aldrich, St. Louis, MO), Cefepime (CPM) (Sigma-Aldrich, St. Louis, MO); Fluoroquinolones: Ciprofloxacin (CIP) (Sigma-Aldrich, St. Louis, MO), Levofloxacin (LEV)(Sigma-Aldrich, St. Louis, MO); Penicillins: Carbenicilin (CB) (Sigma-Aldrich, St. Louis, MO); Penicillins + β-lactamase inhibitors: Piperacillin/Tazobactam (P/T)(MP Biomedicals, Solon, OH); Monobactams: Aztreonam (AZT)(Sigma-Aldrich, St. Louis, MO); Phosphonic acids: Fosfomycin (FOS) (Sigma-Aldrich, St. Louis, MO); and Polymyxins: Colistin (CS)(Sigma-Aldrich, St. Louis, MO). The reference strains used for validation of the techniques were *Pseudomonas aeruginosa* ATCC® 27853 and *Escherichia coli* ATCC® 25922 (American Type Culture Collection, Manassas, VA, USA). The MIC interpretative criteria (μg/ml) was as follows: GEN: S ≤4, I 8, R ≥16; TOB: S ≤4, I 8, R ≥16; AK: S ≤16, I 32, R ≥64; IMI: S ≤2, I 4, R ≥8; MEM: S ≤2, I 4, R ≥8; CAZ: S ≤8, I 16, R ≥32; CPM: S ≤8, I 16, R ≥32; CIP: S ≤1, I 2, R ≥4; LEV: S ≤2, I 4, R ≥8; CB: No reported values; P/T: S ≤16/4, I 32/4-64/4, R ≥128/4; AZT: S ≤8, I 16, R ≥32; FOS: No reported values; CS: S ≤2, I 4, R ≥8. ND: Not determined[18]

### Genotyping via Multilocus Sequence Typing (MLST)

Genotyping of the *P*. *aeruginosa* isolates was performed via MLST of the nucleotide sequences of the genes encoding the following metabolic enzymes: *acs*A (acetyl coenzyme A synthetase), *aro*E (shikimate dehydrogenase), *gua*A (GMP synthetase), *mut*L (DNA repair protein), *nuo*D (NADH dehydrogenase I chain C, D), *pps*A (phosphoenolpyruvate synthase) and *trp*E (anthralitesynthetase component I)[19]. Each gene was sequenced in both sense, and the consensus sequence compared with *P*. *aeruginosa* sequences was registered in the public MLST database [http://pubmlst.org/paeruginosa/] to be assigned an allelic number. The allelic profile was obtained through independent alignment of the pair of sequences for each gene for each strain using ClustalW[20][http://www.clustal.org/clustal2/]. Manual editing of the alignment was performed with Seaview[21][http://pbil.univ-lyon1.fr/software/seaview.html] and FinchTV ver.1.4.0 [http://www.softpedia.com/get/Science-CAD/FinchTV.shtml]. The alleles or STs that did not match any of the existing alleles or STs in the database were designated as new. The new alleles of each different gene (locus) and the new STs were sent to the MLST database curator for addition to the *P*. *aeruginosa* MLST website [http://pubmlst.org/paeruginosa/].

Furthermore, the nucleotide sequences of each gene were translated into amino acid sequences to determine certain variability parameters, such as the ratio of nonsynonymous to synonymous substitutions, the sites of mutational changes, polymorphisms, G+C content, nucleotide diversity (π) and the average number of nucleotide differences (θ). These parameters were obtained using DnaSP ver5.10.01[22][http://www.softpedia.com/get/Science-CAD/DnaSP.shtml].

### Genetic relationships

To identify groups of related STs, we compared the STs identified in this study with those deposited in the MLST *P*.*aeruginosa* database (2266 STs until April 2016) using the eBURST v3 algorithm[23][http://eburst.mlst.net/v3/enter_data/single/]. All members assigned to the same group shared identical alleles at six of the seven loci with at least one other member of the group. Default settings were used to achieve the most stringent definition. Clonal complexes (CC) were defined as the set of STs that descended from the same founding genotype[23].

To provide a graphical representation of the evolutionary relationships between the STs identified in this study and possible events of recombination, a phylogenetic network was built based on the sequences of each locus and the concatenated sequences of seven housekeeping genes of *P*.*aeruginosa* using the neighbor-net algorithm (distance-based method) [24] implemented in SplitsTree ver. 4.0[25]. The reliability of the network was estimated with a Bootstrap test after 1000 pseudoreplicates. The pairwise homoplasy index (PHI) test allowed inference of recombination events during the generation of allelic variation.

## Results

### Bacterial isolates

A total of 58 isolates from 46 patients were obtained during the period from 2007–2013. Among these strains, 65.52% (n = 38) were isolated from urine, 22.41% (n = 13) from blood, 8.62% (n = 5) from a catheter and 3.45% (n = 2) from other sources. These strains were isolated from the following areas: nephrology (20.68%), emergency (15.51%), surgical therapy (15.51%), pediatrics (10.34%), surgery (8.62%),intensive care unit (8.62%), internal therapy (5.17%), oncology (5.17%), neurology (5.17%), cardiology (3.45%) and internal medicine (1.72%). The bacterium was responsible for a mortality rate of 17.39% (for 21.74% of the patients the outcome was unknown).

### Susceptibility profile

The strains were classified as: sensitive (S), resistant (R), multidrug resistant (MDR), extremely drug resistant (XDR) or pan drug resistant (PDR) to different antibiotics according to CLSI guidelines (2016)[18].

The characteristics of each strain of *P*. *aeruginosa* isolated from pediatric patients are shown in Table 1. The antimicrobial susceptibility profile showed that ten strains were sensitive; four were resistant to multiple antibiotics; 42 were extremely resistant; and two were panresistant. Coexistence of S, MDR, XDR and PDR isolates with the same sequence type was noted (Table 1).

**Table 1:** Antimicrobial susceptibility and classification of *P*.*aeruginosa* strains as PDR, XDR, MDR or S.

**P**	**ID**	**Y**	**R**	**S**	**O**	**ST**	**Antibiotics tested**														**RP**
							**1**			**2**		**3**		**4**		**5**	**6**	**7**	**8**	**9**	
							GEN	TOB	AK	IMI	MEM	CAZ	CPM	CIP	LEV	CB	P/T	AZT	FOS	CS	
1	HIM1	2007	S	B	↑	1723	R	ND	R	R	R	R	R	R	ND	> 128	R	R	> 128	I	XDR
2	HIM2/1	2007	STx	C	**†**	1724	ND	ND	R	R	R	R	R	R	ND	> 128	I	R	> 128	S	XDR
	HIM3					1725	R			ND		ND								I	
3	HIM3/1	2007	E	U	↑	1725	R	ND	R	R	R	R	R	R	ND	> 128	R	R	> 128	I	XDR
4	HIM3/2	2007	STx	U	↑	1725	R	ND	R	R	R	R	R	R	ND	> 128	I	R	> 128	I	XDR
5	HIM3/3	2007	N	C	↑	1725	ND	ND	R	ND	R	ND	R	R	ND	> 128	R	R	> 128	I	XDR
	HIM3/4																				
6	HIM3/5	2007	PICU	U	↑	1725	R	ND	R	R	R	R	R	R	ND	> 128	I	R	> 128	I	XDR
7	HIM2	2008	S	B	↑	1724	R	R	R	R	R	R	R	R	R	> 128	I	R	> 128	I	XDR
8	HIM3/6	2008	P	U	↑	1725	R	R	R	R	R	R	R	R	R	> 128	R	R	> 128	I	XDR
9	HIM3/7	2008	S	U	↑	1725	R	R	R	R	R	R	R	R	R	> 128	R	R	> 128	I	XDR
10	HIM3/11	2008	E	U	**†**	1725	ND	ND	I	ND	R	ND	R	R	ND	> 128	I	R	> 128	S	MDR
	HIM3/12						R	R	R	R	R	R	R	R	R	> 128	I	R	> 128	S	XDR
11	HIM3/13	2008	Neph	U	↑	1725	R	R	R	R	R	R	R	R	R	> 128	R	R	> 128	I	XDR
12	HIM3/14	2008	E	U	↑	1725	R	R	R	R	R	R	R	R	S	> 128	R	R	> 128	I	XDR
13	HIM3/15	2008	S	U	↑	1725	R	R	R	R	R	R	R	R	R	> 128	R	R	> 128	I	XDR
14	HIM3/26	2008	Neph	U	↑	1725	S	S	S	S	S	S	S	S	S	> 128	I	R	> 128	S	MDR
15	HIM4	2008	PICU	B	**†**	1726	R	R	R	R	R	R	R	R	R	> 128	R	R	> 128	S	XDR
16	HIM9	2008	E	B	↑	1731	S	S	S	S	S	S	I	S	I	> 128	S	S	> 128	S	S
17	HIM10	2008	PICU	B	**†**	2226	S	S	S	S	S	S	S	S	S	> 128	S	S	128	S	S
18	HIM11	2008	NICU	B	?	1733	S	S	S	S	R	S	S	S	S	> 128	S	S	> 128	I	S
19	HIM12	2008	P	B	↑	1734	S	S	S	S	S	S	S	S	S	128	S	S	> 128	I	S
20	HIM13	2008	O	B	**†**	1737	S	S	S	S	S	S	S	S	S	64	S	S	> 128	I	S
21	HIM14	2008	P	U	↑	1735	S	S	S	S	S	S	S	S	S	64	S	S	> 128	I	S
22	HIM5	2008	P	B	?	1727	R	S	R	R	R	R	R	R	I	> 128	R	R	> 128	I	XDR
	HIM6					1728	ND	ND		ND		ND			ND		I			S	
23	HIM16	2008	Neph	U	↑	1736	S	S	S	S	S	S	S	S	S	128	S	I	> 128	I	S
	HIM16/1		P								I					32		S			
**P**	**ID**	**Y**	**R**	**S**	**O**	**ST**	**Antibiotics tested**														**RP**
							**1**			**2**		**3**		**4**		**5**	**6**	**7**	**8**	**9**	
							GEN	TOB	AK	IMI	MEM	CAZ	CPM	CIP	LEV	CB	P/T	AZT	FOS	CS	
24	HIM7	2009	PICU	B	**†**	1729	R	R	R	R	R	R	R	R	R	> 128	R	S	64	I	XDR
25	HIM3/8	2008	S	U	↑	1725	ND	ND	R	ND	R	ND	R	R	ND	> 128	R	R	> 128	I	XDR
	HIM3/9		Neph																		
	HIM3/10	2010	STx			1730	R	R		R		R			R						
	HIM8		Neph																		
26	HIM15	2008	E	B	**†**	561	S	S	S	S	S	S	S	S	S	> 128	S	S	> 128	I	S
	HIM3/20	2010	0	U		1725	R	R	R	R	R	R	R	R	R		R	R			XDR
27	HIM3/18	2010	STx	U	↑	1725	R	R	R	R	R	R	R	R	R	> 128	R	R	> 128	I	XDR
	HIM3/19						ND	ND		ND		ND			ND						
28	HIM3/16	2010	Neph	U	↑	1725	R	R	R	R	R	R	R	R	R	> 128	R	R	> 128	S	XDR
29	HIM3/17	2010	STx	U	↑	1725	R	R	R	R	R	R	R	R	R	> 128	R	R	> 128	S	XDR
30	HIM3/21	2010	Neph	C	↑	1725	R	R	R	ND	R	R	R	R	R	> 128	R	R	> 128	I	XDR
31	HIM3/22	2010	STx	B	↑	1725	R	R	R	R	R	I	R	S	S	> 128	R	R	> 128	I	S
	HIM3/23			U								R		R	R					S	
32	HIM3/24	2010	ITx	U	↑	1725	R	R	R	R	R	R	R	R	R	> 128	R	R	> 128	I	XDR
	HIM3/25																				
33	HIM20	2011	Neph	M	?	2246	R	R	R	R	R	R	R	R	R	> 128	I	R	> 128	I	XDR
34	HIM17	2012	N	U	↑	2243	R	R	R	R	R	R	R	R	R	> 128	R	R	> 128	I	XDR
35	HIM18	2012	O	U	↑	2244	R	R	S	R	R	R	R	R	R	> 128	R	R	> 128	I	XDR
36	HIM19	2012	ITx	U	?	2245	R	R	R	R	R	R	R	R	R	> 128	I	R	> 128	I	XDR
37	HIM3/27	2012	E	U	↑	1725	R	R	R	R	R	R	R	R	R	> 128	R	R	> 128	I	XDR
38	HIM3/28	2012	Neph	U	↑	1725	R	R	R	R	R	R	R	R	R	> 128	I	R	> 128	R	XDR
39	HIM3/29	2013	E	U	↑	1725	R	R	R	R	R	R	R	R	R	> 128	R	R	> 128	R	PDR
40	HIM3/30	2013	Neph	U	?	1725	R	R	R	R	R	R	R	R	R	> 128	R	R	> 128	I	XDR
41	HIM3/31	2013	Neph	U	?	1725	R	R	R	R	R	R	R	R	R	> 128	R	R	> 128	I	XDR
42	HIM3/32	2013	C	U	?	1725	R	R	R	R	R	R	R	R	R	> 128	R	R	> 128	R	PDR
43	HIM22	2013	C	M	**†**	2248	R	R	S	R	R	R	R	S	R	> 128	I	R	> 128	S	MDR
44	233	2013	E	U	?	233	R	R	R	R	R	R	R	R	R	> 128	R	S	64	R	XDR
45	233/1	2013	Neph	U	?	233	R	R	R	R	R	R	R	R	R	> 128	R	S	> 128	I	XDR
46	112	2013	Imed	U	?	112	R	R	R	I	R	R	R	S	S	> 128	S	S	> 128	R	MDR

P: Patient; ID: Assigned in the MLST *P*. *aeruginosa* database; Y: Year; R: Room; S: Source; O: Outcome; ↑: Discharged; **†:** Dead;?: Unknown; ST: Sequence Type; B: Blood; C: Catheter; U: Urine; M: Miscellaneous; S: Surgery; STx: Surgical Therapy; E: Emergency Room; N: Neurology; PICU: Pediatric Intensive Care Unit; P: Pediatrics; Neph: Nephrology; ITx: Internal Therapy; O: Oncology; C: Cardiology; NICU: Neonatal Intensive Care Unit; IMed: Internal Medicine; Antibiotics tested: Aminoglycosides: Gentamicin (GEN), Tobramycin (TOB), Amikacin (AK); Carbapenems: Imipenem (IMI), Meropenem (MEM); Cephalosporins: Ceftazidime (CAZ), Cefepime (CPM); Fluoroquinolones: Ciprofloxacin (CIP), Levofloxacin (LEV); Penicillins: Carbenicilin (CB); Penicillins + β-lactamase inhibitors: Piperacillin/Tazobactam (P/T); Monobactams: Aztreonam (AZT); Phosphonic acids: Fosfomycin (FOS); Polymyxins: Colistin (CS). (CLSI, 2016) [18]

PDR: Pan Drug Resistant. XDR: Extensively Drug Resistant. MDR: Multi Drug Resistant. S: Sensitive. Reference strains: *P*. *aeruginosa* ATCC® 27853 and *Escherichia coli* ATCC® 25922.

Discharge/d patients: 60.87%. Mortality rate: 17.39%. Unknown patient outcome: 21.74%.

### MLST

MLST analysis identified 23 different STs among the 58 *P*. *aeruginosa* strains (Table 2): ST1725 was detected in 33 strains from 26 patients (two PDR, two MDR, 28 XDR and one susceptible); two XDR strains from two patients contained ST1724; two strains from the same patient contained ST1736; two strains from two patients contained ST233 (XDR); and the remain strains (19) from 18 patients presented a distinct ST. Three patients exhibited coexistence of two different ST´s at the same year (Patient two, 22, and 25) (Table 1). Twenty-one new STs of *P*. *aeruginosa* were obtained. The first STs reported in Mexico are shown in Table 2, and only two of the STs obtained in this study were previously reported (ST233 and ST112).

The greatest diversity of alleles was found in the *trpE* (12) gene, followed by *guaA*(11), *acsA* and *ppsA* (10), *aroE* (9), *mutL* (8) and finally, the *nuoD* (5) gene.

**Table 2:** Allelic profiles and relevant information for the identified STs.

ID	ST	*acs*A	*aro*E	*gua*A	*mut*L	*nuo*D	*pps*A	*trp*E	CC	Relevant characteristics
HIM1	1723	13	91	9	3	1	17	15	CC309	**NST**, XDR
HIM2	1724	38	91	3	9	1	2	4	CC235	**NST**, XDR (two isolates)
HIM3	1725	13	52	9	3	1	17	15	CC309	**NST**, PDR, XDR, MDR, S (33 isolates)
HIM4	1726	41	91	3	9	1	2	4	CC235	**NST**, XDR
HIM5	1727	38	91	9	9	1	2	8	Singleton	**NST**, XDR
HIM6	1728	38	91	3	9	1	2	26	CC235	**NST**, XDR
HIM7	1729	4	75	16	12	1	18	3	CC253	**NST**, XDR
HIM8	1730	13	52	9	3	12	17	15	CC309	**NST**, XDR
HIM9	1731	11	5	1	25	1	15	1	Singleton	**NST**, S
HIM10	2226	38	52	3	33	1	18	183	Singleton	**NST**, S
HIM11	1733	5	154	26	21	24	33	42	Singleton	**NST**, S
HIM12	1734	11	10	6	11	4	4	7	Singleton	**NST**, S
HIM13	1737	39	80	12	11	1	15	2	CC245	**NST**, S
HIM14	1735	6	5	5	3	3	4	26	CC1025	**NST**, S
HIM15	561	39	80	12	11	3	15	2	CC245	**NST**, S
HIM16	1736	17	78	12	3	1	1	18	CC931	**NST**, S (two isolates)
HIM17	2243	13	52	3	3	1	17	15	CC309	**NST**, XDR
HIM18	2244	13	52	9	10	1	17	15	CC309	**NST**, XDR
HIM19	2245	13	17	9	3	1	17	15	CC309	**NST**, XDR
HIM20	2246	13	75	5	10	12	7	15	CC308	**NST**, XDR
HIM22	2248	13	5	9	3	1	33	1	Singleton	**NST**, MDR, Singleton
	233	16	5	30	11	4	31	41	CC233	PDR (in this work, 2013), XDR (in this work, 2013), UK 2005, Poland 2005, Norway 2006, Nigeria 2009, Australia 2002, Ivory Coast 2011, France 2009, 2014, Egypt 2015[26].
	112	6	5	1	25	1	12	1	CC395	XDR (in this work, 2013), UK 2003

ID: Assigned in the MLST *P*. *aeruginosa* database; ST: Sequence Type; CC: Clonal Complex; **NST: New Sequence Type**; PDR: Pan Drug Resistant; XDR: Extensively Drug Resistant; MDR: Multi Drug Resistant; S: Sensitive.

Data for ST233 and ST112 was taken from the public MLST database [http://pubmlst.org/paeruginosa/] and Zafer et al., 2015.

### Nonsynonymous and synonymous substitutions

The *pps*A and *trp*E genes showed nonsynonymous substitutions, while the *gua*A, *mut*L and *nuo*D genes presented synonymous substitutions. The *acs*A and *aro*E genes exhibited both nonsynonymous and synonymous substitutions. The codons that most frequently presented synonymous changes was alanine, followed by serine, valine, glycine, proline and lysine; the codons that most frequently presented nonsynonymous changes were serine (TCT) to proline (CCT), serine (TCT) to alanine (GCT), serine (AGT) to asparagine (AAT) and serine (TCT) to phenylalanine (TTT), threonine (ACT) to alanine (GCT), threonine (ACC) to isoleucine (ATC), threonine (ACG) to alanine (GCG),and histidine (CAC) to tyrosine (TAC). A total of 71 changes occurred in all the genes, 36 of which were nonsynonymous substitutions, while 35 were synonymous (ratio 1:1).

The G+C content ranged between 63% (*nuo*D) and 70% (*aro*E). The number of alleles per gene varied from five (*nuo*D) to 12 (*trp*E). The number of polymorphic sites was low (2.49%) in the concatenated sequences, resulting in low values of π and θ (Table 3). The ratio of nonsynonymous to synonymous substitutions (dN/dS) was 0 for the *gua*A, *mut*L and *nuo*D genes, 0.004 for *acs*A, and 1.32 for *aro*E. The *pps*A and *trp*E genes showed no synonymous substitutions.

**Table 3:** Important characteristics and housekeeping gene substitutions of *P*. *aeruginosa*.

Allele	Size (bp)	H	PS	π	θ	G+C	dN	dS	dN/dS	PHI test
*acsA*	390	11	12	0.00747	0.00715	0.6895	0.00012	0.02961	0.004053	0.08185
*aroE*	498	9	15	0.00674	0.00646	0.7044	0.00731	0.00553	1.321881	0.004564
*guaA*	373	9	8	0.00351	0.0046	0.6592	0	0.01445	0	1.0
*mutL*	442	8	6	0.00254	0.00291	0.6753	0	0.01008	0	0.04163
*nuoD*	366	5	6	0.00114	0.00352	0.6337	0	0.00481	0	n.a.
*ppsA*	370	10	8	0.00475	0.00464	0.6639	0.00665	0	n.a.	0.2692
*trpE*	443	12	17	0.00758	0.00823	0.6644	0.01104	0	n.a.	2.922E-4
Concatenate	2882	64	72	0.00482	0.00536	0.67006	0.00359	0.00921	0.389578	1.73E-13

H: Haplotype; PS: Polymorphic sites; Π: Nucleotide diversity; Θ: Average number of nucleotide differences; G+C: Guanine and cytosine content; dN: Number of nonsynonymous substitutions; dS: Number of synonymous substitutions. PHI test: Statistically significant recombination (p<0.05).

### Genetic relationships

**Fig 1:**
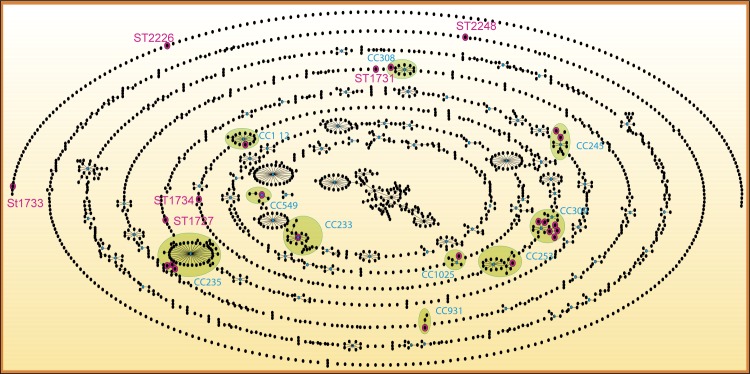
Network of the 2266 sequence types listed in the *Pseudomonas aeruginosa* PubMLST database (April 2016). *P*. *aeruginosa* isolates (n = 59 STs). Black points represent sequence types (STs); lines connect single-locus variants (SLVs). The STs with pink halos were described in this study. Blue points represent founder STs, and the clonal complexes (CC) formed are highlighted in green. The strict definition of group was used (6/7 shared alleles).

**Fig 2:**
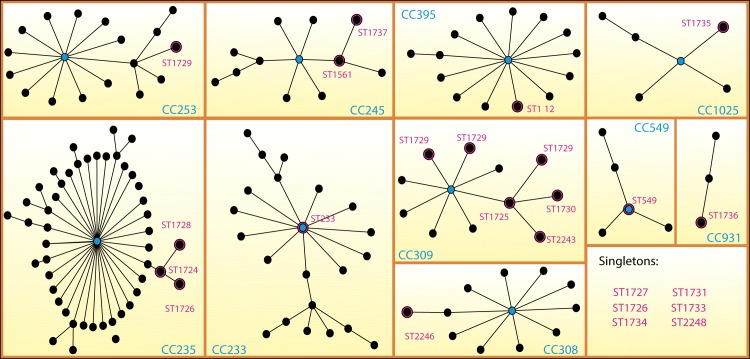
Diagram of clonal complexes of *Pseudomonas aeruginosa* strains produced with the eBURST v3 algorithm. *P*. *aeruginosa* isolates (n = 59 ST). Black points represent sequence types (STs); lines connect single-locus variants (SLVs) or double-locus variants (DLVs). The STs with pink halos were described in this study. Blue points represent founder STs.

The STs identified in this study integrated nine clonal complexes (CC309, CC235, CC253, CC245, CC1025, CC931, CC308, CC233 and CC395) and six singletons (Figs 1 and 2). Clonal complex CC309 consisted of ST1723, ST1725, ST1730, ST2243, ST2244 and ST2245. ST1725 was found in 33 strains and was the sub-founder group. CC235 contained ST1724, ST1726 and ST1728 and was the clonal complex with the second largest number of associated STs (48 STs) throughout the population network in the PubMLST database. ST233 (previously reported in other countries) was the founder group of clonal complex CC233 and shows relevance for strains with XDR characteristics identified in this study. The remaining clonal complexes contained one or two of the STs identified in this study. The MDR and XDR strains were grouped in prevalent STs, but the S strains corresponded mainly to singleton STs, as described by Gomila *et al*. (2013)[27] (Fig 2).

**Fig 3:**
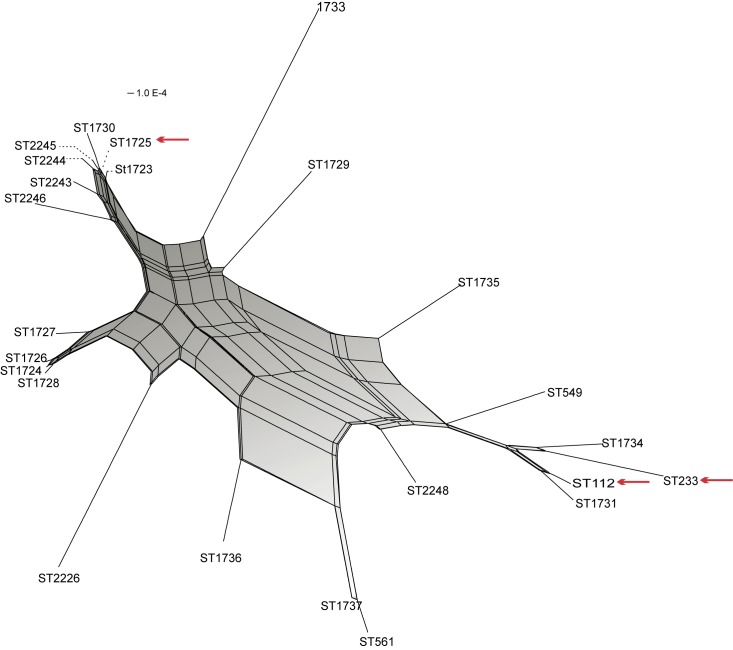
Neighbor-net graph based on the concatenated sequences of seven housekeeping genes of *Pseudomonas aeruginosa* used for MLST analysis. The PHI test detected statistically significant evidence of recombination (p = 1.729E-13).

The genetic relationships between different STs were corroborated with the phylogenetic neighbor-net network, in which four outstanding complexes were observed: a) one integrating ST2246, ST2243, ST2244, ST2245, ST1723, ST1730 and ST1725, which corresponded to CC309 indentified by the eBURST algorithm; b) one integrating ST1727, ST1726, ST1724 and ST1728, identified as CC235; c) one integrating ST549, ST1734, ST112, ST1731 and ST233, identified in different clonal complexes and singletons by the eBURST algorithm; and d) one integrating ST1737 and ST561, which corresponds to CC245 (Figs 1 and 3).

Both analyses, eBURST and Neighbor-net are based on the same set of loci, and therefore not independent from one another.

The phylogenetic network showed a close relationship between CC309 and CC235 and a distant relationship between these clonal complexes and CC233. The short distance between ST112 and ST233 (both STs were previously reported in European countries) is highlighted, although these STs are positioned far away in the diagram of the ST1725 clone. The sequence types that showed the most support were ST1733, ST2226 and ST1729. ST112 and ST233 were positioned very close and were grouped together in the network. ST549 (PAO1) exhibited a short distance from the CC with ST233 as the principal component. ST1723, ST1724 and ST1725 were isolated in 2007 and shared a close relationship. Most of the STs were isolated in 2008, and the distance between them was variable. ST1724 (isolated in 2007) probably gave rise to ST1727, ST1726 and ST1728 (isolated in 2008). STs isolated in 2013 (ST2248, ST112 and ST233) were separated by only a short distance and were grouped at the bottom of the diagram. ST1725 was isolated in 2007, 2008, 2010, 2012 and 2013, showing different resistance characteristics (Fig 3).

The PHI test of the concatenated sequences revealed statistically significant recombination events (p<0.05) (Table 3), which is graphically illustrated in the phylogenetic network by the presence of rectangular and square boxes indicating a high probability of extensive homologous recombination (Fig 3). The PHI test showed statistically significant recombination events in the *aroE*, *mutL* and *trpE* genes, which were not found in the *guaA* and *ppsA* genes. It was not possible to apply PHI analysis to the *nuoD* gene because the number of haplotypes for this gene was small (n = 5) (Table 3).

## Discussion

Our results reveal the identification of different *P*.*aeruginosa* clones among forty-six patients with NIs from 2007 to 2013 at a pediatric tertiary care hospital in Mexico City. These clones vary with respect to their susceptibility to antibiotics, with most showing high resistance profiles. The bacterium was mainly isolated from urine and blood and was responsible for a mortality rate of 17.39% (for 21.74% of the patients the outcome was unknown). High rates of mortality caused by specific *P*. *aeruginosa* strains are of particular global concern, and there have been reports of rates ranging from 42% to 80% in areas such as South Africa[4].

### Susceptibility profile and genotyping

Two of the strains were PDR; 42 were XDR; four were MDR; and ten were S. Twenty-one of the strains exhibited different allelic profiles from those known worldwide, and thirty-two strains showed the same sequence type. The latter finding was of particular relevance because this was a new ST (ST1725) isolated from different hospital areas throughout the study period (2007 to 2013) and from patients with different underlying diseases. This ST1725 clone is highlighted in relation to the other STs because of its higher frequency. Another relevant sequence type was ST233, which was isolated at a low frequency but had been previously reported in other countries as a high-risk clone[28].

In recent years, the antimicrobial resistance of *P*. *aeruginosa* strains has markedly increased. It has been reported that 10.5% of *P*. *aeruginosa* strains isolated from various hospitals in Spain are XDR, exhibiting ST111 and ST175[14]; Gomila *et al*. (2013) reported a rate of 21.4% for XDR strains and 17.2% for MDR strains of *P*. *aeruginosa* (n = 56)[27].

### New clone ST1725 and high-risk clone ST233

Worldwide, there have been reports of STs of high-risk clones with MDR and XDR characteristics. Apparently, these STs have been confined to specific geographical areas; currently the ST111, ST175, ST235, Liverpool, Manchester and Melbourne clones are widely distributed in various hospitals in both developed and undeveloped regions, including Europe, South Africa, Asia and South America[8].

The ST1725 clone reported here was observed in 26 patients. It was first identified in 2007 and continually isolated up to 2013, showing S, MDR and mainly XDR characteristics; however, two strains isolated from different patients in the last year showed PDR characteristics (Table 1). Several authors have described this phenomenon, arguing that resistance accumulates significantly over time due to the ability of some clones to acquire resistance mechanisms or by means of cross-transmission between strains[4,7].

The ST233 clone was identified twice in the present study. The clones were identified, in September 2013, and in October of the same year in different patients, both clones were XDR. Previous reports have identified ST233 as the cause of major outbreaks around the world, including a Japanese case that occurred between 2006 and 2009. More recently, ST233 was identified in a *P*. *aeruginosa* MDR outbreak causing bacteremias in South Africa in 2010–2011[28]; however, the ST233 strains were not found to be resistant to colistin, the antibiotic that has been used as the treatment of last resort for XDR *P*. *aeruginosa* outbreaks, in any of these cases[12]. Thus, this represents the first report of an ST233 strain with XDR characteristics. This clone was resistant to colistin, but aztreonam susceptible. Multiple reports warn about the presence of significant *P*. *aeruginosa* STs and highlight the presence of MDR and XDR clones that are susceptible to only colistin[29], with two exceptions: the ST244 strain from Korea [30] and the ST348 strain from Spain[13], both of which have been reported to be resistant to colistin, but sensitive to other antibiotics. Our results draw attention to some STs reported as XDR that present MIC values for colistin of up to 16 μg/mL.

### Nonsynonymous and synonymous substitutions

The G+C content identified in this study is similar to that reported in previous studies[3]. The estimated number of alleles per gene matches the number of alleles per gene reported in the MLST database, where the *nuoD* gene has 103 alleles, while the *trpE* gene has 200, which is only surpassed by the *aroE* gene, with 206 alleles.

Furthermore, the *ppsA and trpE* genes presented only nonsynonymous substitutions, while the *guaA*, *mutL* and *nuoD* genes presented only synonymous substitutions, and the *acsA* and *aroE* genes showed both types of substitutions. The dN/dS ratio of *acsA* and *aroE* means that the first gene is evolving apparently free from natural selection (*P*>0.05) and the second across purifying selection (*P<* 0.05) that suggest that the probability of fixation of new mutants is lower, and if they are present, their frequency decreases in subsequent generations [31].

### Genetic relationships

The PHI test and the neighbor-net phylogenetic network based on the concatenated genes suggested recombination events between strains isolated in the hospital with an overrepresentation of certain STs, which is consistent with findings reported by other authors [3,11,27]. A non-clonal epidemic population structure is characterized by a reduced number of abundant genotypes and some CCs consisting of three or more STs[3,11]. Our results reveal frequent recombination between housekeeping genes, particularly the *aroE*, *mutL* and *trpE* genes, and clearly show a non-clonal epidemic population structure. It was not possible to apply the PHI test for the *nuoD* gene. Nevertheless, no recombination events were graphically illustrated, with a tree-like structure being obtained, indicating that this gene is not affected by intergenic recombination, as found in the phylogenetic structure obtained for the *guaA* and *ppsA* genes. It should be noted that at least three major STs, ST1725, ST233 and ST1724, form a relevant CC due to their frequency and association with other STs.

The 23 STs found in the present study are grouped into nine CCs, among which CC309, CC235 and CC233 are highlighted. CC309 mostly consists of new STs identified in this study and has not been reported previously, whereas CC235 and CC233 have already been widely described and are considered to belong to the clones of high epidemic risk[8].

Although we did not find ST235 in the present study, three STs (ST1724, ST1726 and ST1728) associated with the CC led by ST235 were identified, which suggests that they may be dangerous variants that retain the characteristics of XDR, with possible properties that allow them to spread. CC235 has 48 different associated STs and is the second largest CC in the network constructed with eBURST. The possibility that the ST1725 clone (first described in this study) from CC309 may exhibit the characteristics and mechanisms necessary to be a high-risk clone with XDR and PDR characteristics and cause death is not discarded.

### Successful clones

It is widely accepted that antimicrobial susceptibility is not confined to a single genetic lineage or subpopulation [13]; strains with the same ST show different susceptibilities, and strains with different STs sometimes show the same or similar susceptibilities. However, the most successful clones are more likely to acquire MDR determinants and are therefore more easily selected under pressure from various antibiotics, which contributes to their persistence and spreading in hospitals[12,14].

The transmission of high-risk ST clones is still poorly understood in many ways. It has been observed that some of the most prevalent STs are frequently found as environment pollutants[27], and it is assumed that immunocompromised patients are able to acquire the bacteria directly from the environment, or possibly from inanimate fomites, or even from medical staff[12]. The present study suggests interpatient propagation of clones[14] that already possessed outstanding characteristics of resistance (XDR, ST1725). Other studies support the notion that spreading of interpatient mutant lineages occurs after the acquisition of resistance mechanisms[7]. Furthermore, Cabot *et al*. (2012) [15]reported greater clonal diversity among multisensitive strains, a finding consistent with the results of our study.

In previous studies no statistically significant differences among the variables resistance to antibiotics, sex, age, origin of the simple, area of the hospital or ST were observed, the authors attributed this to the small sample size[27]. However, in our study, a relationship was observed between the acquisition of resistance over time and the coexistence of sensitive and resistant strains with the same ST (Table 1).

Whereas many STs have succeeded for years, the existence of recombination events within microbial populations suggests that strains and CCs are transient[11]. At the HIMFG, ST1725 has remained for at least seven years, which is indicative of its great stability and adaptive capacity. This represents a potential danger to the hospital and indicates the possibility of explosive spreading to different sites.

### Contributions

This study is relevant because it identifies the presence of the ST1725 and ST233in Mexico for the first time. While the latter clone has previously been described in other countries, the most concerning finding of this study is that both STs are shown to have XDR characteristics, ST1725 PDR characteristics for first time. Despite high levels of genetic diversity, ST1725 could be associated with the multiresistant phenotype.

As in other studies, it is difficult to identify the source of the bacteria, and a prospective study including a deliberate search among different areas of the hospital where there have been recent cases of nosocomial infections with *P*. *aeruginosa* would therefore be beneficial. Since 2014, the HIMFG has implemented a program called “Let´s go for the 100”, which aims to reduce nosocomial infections through continuous and constant sanitization of the hospital and proper cleaning, care and hygiene of all medical personnel. The results have been reflected in a significant reduction in the isolation of ST1725 and ST233. However, surveillance and monitoring of these STs continue.

In conclusion, the identification of some STs was detected in 2007, after which recombination events occurred and produced significant diversity, which was detected in 2008. Nevertheless, ST1725 has been strongly selected and has persisted over the years, giving rise to certain variants in 2012, but none have been more successful than ST1725. Thus, we consider it important to continue monitoring and characterizing this clone because of its great potential to be a novel high-risk clone.
